# Responsiveness to change for the Brazilian Scale of Oral Health Outcomes for 5-year-old children (SOHO-5)

**DOI:** 10.1186/1477-7525-11-137

**Published:** 2013-08-09

**Authors:** Jenny Abanto, Georgios Tsakos, Thiago Machado Ardenghi, Saul Martins Paiva, Daniela Prócida Raggio, Aubrey Sheiham, Marcelo Bönecker

**Affiliations:** 1 Department of Pediatric Dentistry and Orthodontics, Faculty of Dentistry, University of São Paulo, Av. Lineu Prestes 2227, 05508-000, São Paulo, SP, Brazil; 2 Department of Epidemiology and Public Health, University College London 1–19 Torrington Place, WC1E 6BT, London, UK, England; 3 Department of Stomatology, Dental School, Federal University of Santa Maria, Santa Maria, Brazil; 4 Department of Pediatric Dentistry and Orthodontics, Faculty of Dentistry, Federal University of Minas Gerais, Av. Antônio Carlos 6627, 31270-901, Belo Horizonte, MG, Brazil

## Abstract

**Background:**

The responsiveness of oral health-related quality of life (OHRQoL) instruments has become relevant, given the increasing tendency to use OHRQoL measures as outcomes in clinical trials and evaluations studies. The purpose of this study was to assess the responsiveness of the Brazilian Scale of Oral Health Outcomes for 5-year-old children (SOHO-5) to dental treatment.

**Methods:**

One hundred and fifty-four children and their parents completed the child self- and parental’ reports of the SOHO-5 prior to treatment and 7 to 14 days after the completion of treatment. The post-treatment questionnaire also included a global transition judgment that assessed subject’s perceptions of change in their oral health following treatment. Change scores were calculated by subtracting post-treatment SOHO-5 scores from pre-treatment scores. Longitudinal construct validity was assessed by using one-way analysis of variance to examine the association between change scores and the global transition judgments. Measures of responsiveness included standardized effect sizes (ES) and standardized response mean (SRM).

**Results:**

The improvement of children’s oral health after treatment are reflected in mean pre- and post-treatment SOHO-5 scores that declined from 2.67 to 0.61 (p < 0.001) for the child-self reports, and 4.04 to 0.71 (p < 0.001) for the parental reports. Mean change scores showed a gradient in the expected direction across categories of the global transition judgment, and there were significant differences in the pre- and post-treatment scores of those who reported improving a little (p < 0.05) and those who reported improving a lot (p < 0.001). For both versions, the ES and SRM based on change scores mean for total scores and for categories of global transitions judgments were moderate to large.

**Conclusions:**

The Brazilian SOHO-5 is responsive to change and can be used as an outcome indicator in future clinical trials. Both the parental and the child versions presented satisfactory results.

## Introduction

Dental caries and complicated traumatic dental injuries (TDI) have a negative impact on oral health-related quality of life (OHRQoL) in young children [1-5]. In Brazil, the prevalence of untreated dental caries is high (80%) at 5 years old according to the latest national epidemiological survey [6], whereas TDI is an emerging and challenging, but mostly overlooked, public health problem [7]. Consequently, it is important to assess the effectiveness of clinical interventions to treat these conditions, using quality of life as one of the key outcomes.

The Scale of Oral Health Outcomes for 5-Year-Old Children (SOHO-5) was recently developed to assess the Oral Health Related Quality of Life (OHRQoL) in young children through both self-reports and proxy-reports [8]. Its cross-sectional validity, test-retest reliability and reproducibility properties were tested in previous studies [8,9]. Nevertheless, in order to be used as an outcome for the evaluation of an intervention, an instrument needs additional properties to detect minimally important clinical changes after treatment. This latter property is known as “responsiveness” [10]. Responsiveness is a key technical property, which assists researchers to choose the most appropriate measure for clinical trials, provide a basis for estimating sample sizes, and facilitates interpretation of the change scores after treatment derived from the measures [11,12]. To date, the responsiveness of OHRQoL instruments has become relevant, given the increasing tendency to use OHRQoL measures as outcomes in clinical trials and evaluations studies. However, there are still only a handful of studies on responsiveness for schoolchildren’s OHRQoL instruments [13-16] and scarce for preschool children [17,18]. Besides that, at present, the responsiveness of the SOHO-5, which is the only instrument available to assess child-self reports concerning OHRQoL, has not been established.

The assessment of responsiveness also allows the calculation of the minimal important differences (MID) [10,19]. The MID is defined as ‘the smallest difference in score, which patients perceive as beneficial and which would mandate, in the absence of troublesome side-effects and excessive cost, a change in the patient’s management’ [20]. In essence, the MID provides a good indication of whether the observed change is meaningful. Consequently, an intervention may be justified if it can be shown to result in a change that exceeds the MID [21]. That is a stronger reason to the MID be assessed together the responsiveness.

This study aimed to investigate the responsiveness of the Brazilian language version of the SOHO-5 to dental treatment.

## Methods

This study was independently reviewed and approved by the Human Research Ethics Committee of the Dental School of the University of São Paulo.

### Study population

There are no guidelines regarding the sample size needed for a study of responsiveness [22]. However, the sample size was calculated considering the following parameters: 5% of standard error, 80% of power, a confidence level of 95%, mean scores for the pre-treatment questionnaires equal to 5.95 (±sd:3.10) and 10.79 (±sd: 6.81) for the child and parental version, respectively. The mean scores for the post-treatment questionnaires were 3.45 (±sd:2.74) for the child-self report version and 4.51 (±sd:4.78) for the parental version. These values were based in a previous study that validated the Brazilian version of the SOHO-5 [9]. Taking into consideration a possible lost of 30% during the follow-up, the minimum sample size that would be necessary to satisfy these requirements were estimated in 58 parents and 37children for the child self-reported and parental version, respectively.

Data were collected from 5-6-year-olds children and their respective parents who attended for dental screening at the Dental School, University of São Paulo (USP), Brazil, in 2011. All children and parents were invited to participated in the study, according to the following inclusion criteria: children who had not undergone dental treatment in the last three months, with no systemic diseases, with parents fluent in Brazilian Portuguese and who were willing to participate in the study.

### Study procedures

On the same day of the dental screening, the child and one of the parents completed the Brazilian SOHO-5 in face-to-face independent interviews [9]. The interviews were conducted on the same day prior to the clinical examinations by four trained interviewers who were blind to the clinical findings. In addition, parents answered a question on family income. Seven to 14 days after the completion of treatment [13,17], the interviewer repeated a follow-up SOHO-5 to all those participating on the day of dental screening. In addition, a question to assess perceived change in children’s oral health since the completion of treatment was done. Dentists that conducted the treatments were blinded to the participant’s responses to the SOHO-5 questions.

### Measure

#### Oral health-related quality of life

The Brazilian SOHO-5 considers the child’s lifetime experience of oral impacts in children and parent’s responses [8,9]. It contains two versions of 7 items in each version (child and parental), with 6 of them being common in terms of content.

The 7 items of the child-self report version are: difficulty eating, difficulty drinking, difficulty speaking, difficulty playing, difficulty sleeping, avoid smiling (due to pain) and avoid smiling (due to appearance). Answers are given through a 3-point scale (no = 0, a little = 1, a lot = 2), aided by a prompt/explanation card with appropriate faces.

The 7 items of the parental version are: difficulty eating, difficulty speaking, difficulty playing, difficulty sleeping, avoid smiling (due to pain), avoid smiling (due to appearance), and affected self-confidence of the child. These are answered on a 5-point scale (no = 0, a little = 1, moderate = 2, a lot = 3, a great deal = 4).

The total SOHO-5 score for each version is calculated as a simple sum of the response codes. Since there were 7 questions, the final score can vary from 0 to 14 for the child and from 0 to 28 for the parental version. A higher score denotes a greater degree of oral impacts on the quality of life of the child.

#### Global transition judgments

Participant’s perceptions of change in their oral health since the completion of treatment at the clinic were assessed by a single item with a 5-point response scale (‘Worsened a lot’; ‘Worsened a little’; ‘Stayed the same’; ‘Improved a little’; ‘Improved a lot’). Such transition judgments are often used as a ‘gold standard’ when evaluating the sensitivity to change of health-related quality of life measures [19,23,24]. One advantage of these judgments appears to be that they are not affected by an individual’s mood [25].

### Statistical analysis

#### Responsiveness

Change scores for the scale were calculated by subtracting post-treatments scores from pretreatment scores [13-18,24]. Consequently, positive change scores indicate an improvement in OHRQoL, while negative scores indicate deterioration. The responsiveness was assessed in different ways. The standardized effect sizes (ES) [26] and the standardized response mean (SRM) [27] have been widely recommended and used today as indicators of responsiveness. Both provide direct information on the magnitude of change in the measure, expressed in terms of some measure of variation in the change scores. The ES and SRM are calculated as ratios of the mean change score with the standard deviation of the baseline score and the change score, respectively. Both ES and SRM are expressed in standard deviation units and can be interpreted through conventional benchmarks [26] as indicating small (≤ 0.2), moderate (0.3–0.7) or large (≥ 0.8) effect.

Following the approach of previous studies [14,24,28], paired t-tests were used to examine the significance of the within-subject change of those who changed and those who reported stability. If a measure is responsive, the former should be significant and the latter nonsignificant.

The MID should be calculated through different methods. For distribution-based methods, this implies calculating ES and SRM. For anchor-based methods, this implies the global transition judgments for each version of the SOHO-5 using the mean change scores of those reporting that they “improved a little” in overall quality of life after treatment [19]. This value was also used to calculate Guyatt’s responsiveness statistic [10]. The most appropriate indicator of responsiveness relates to the variability in test scores in stable subjects to the clinically important difference [10]. Consequently, the index is given by the MID divided by the standard deviation of change scores for stable subjects.

#### Longitudinal construct validity

The longitudinal construct validity was assessed by using one-way analysis of variance to examine the association between change scores and the global transition judgments collected pos-treatment. Given the method of calculating change scores, good longitudinal construct validity is indicated if those reporting deterioration have negative mean change scores, those reporting stability have change scores close to zero, and those reporting improvement have positive change scores of increasing magnitude [22,24].

## Results

### Response and characteristics of participants

All the participants interviewed in this study completed all the items of the Brazilian SOHO-5, and no questionnaires were excluded from data analysis due to missing data. The pretreatment SOHO-5 was completed by 193 children and their parents, of whom 154 (79.8%) also completed the post-treatment SOHO-5. There were no statistically significant differences between the subjects who were lost in the follow-up from those who remained in the trial regarding baseline variables such as family income, gender, prevalence of impacts (SOHO-5 score > 0) and age. Descriptive sociodemographic and clinical data for the sample are shown in Table 1. Global transition judgements from children and parental reports reported higher gradient of improvement of the children’s oral health after treatment, however, only 3.9% and 6.5%, for child and parental reports, respectively, reported no change. There were no patients reporting any level of deterioration. The large majority of the sample received operative treatment (71.4%), mainly focused on dental caries treatment (55.2%).

**Table 1:** Sociodemographic and clinical data of the sample (N = 154)

**Variables**	**Category**	**N**	**%**
**Age groups**	5 years-old	89	57.8
	6 years-old	65	42.2
**Gender**	Boy	81	52.6
	Girl	73	47.4
**Caries severity**	Caries free	69	44.8
	Low severity (def-t =1-5)	45	29.2
	High severity (def-t ≥ 6)	40	26.0
**Trauma severity**	Absent	103	66.9
	Uncomplicated trauma	26	16.9
	Complicated trauma	25	16.2
Household income*	< 1 BMW	43	27.9
	≥ 1 BMW	111	70.1
**Type of received treatment**	With treatment (Operative)	110	71.4
	Without treatment (Preventive)	44	28.6
**Global transition judgement of children self reports**	No change	6	3.9
	Improved a little	24	15.6
	Improved a lot	124	80.5
**Global transition judgement of parental reports**	No change	10	6.5
	Improved a little	33	21.4
	Improved a lot	111	72.1

*BMW = Brazilian Minimum Wage in 2011 (approximately $350 during the data gathered).

def-t = decayed, indicated for extraction owing to caries and filled primary teeth.

### Responsiveness

Based on the global transition judgments results, the vast majority of children improved a little or a lot their OHRQoL in a higher frequency than those who did not change. These changes are reflected in mean pre- and pos-treatment SOHO-5 scores that declined from 2.67 to 0.61 (p < 0.001) for the child-self reports, and 4.04 to 0.71 (p < 0.001) for the parental reports (Table 2). These declining change scores indicated an improvement in children’s OHRQoL. For child-self-report version, the ES and SRM for change total scores means showed to be moderate (0.7) and large (0.8), respectively, whereas for the parental version both indicators of responsiveness were moderate (0.6 and 0.7, respectively) (Table 2). These are estimates of the average treatment effect. Table 2 also shows ES and SRM of the SOHO-5 for each category of the global transition judgment. Regarding the child-self report version, ES and SRM for all those who reported improvement were moderate to large, as well as for those who reported stability. The small number of subjects reporting no change means that the estimates for this category should be treated with caution. Regarding the parental version, the ES and SRM for all categories of global transition judgments were moderate with higher values for those that improved a lot compared to the other categories.

**Table 2:** Mean change scores for total scores and within-group comparisons for total scores before and after treatment by global transition judgement (n = 154)

	**Range in baseline**	**Baseline mean (SD)**	**Follow-up mean (SD)**	p- value*	**Mean change scores (SD)**	**ES**	**SRM**
**Child- Self report version**							
Total score	0-12	2.7(3.2)	0.6(1.1)	<0,01	2.1 (2.6)	0.7	0.8
No change		0.2(0.4)	0.5(0.8)	0.36	−0.3(0.8)	0.8	0.4
Improved a little		2.8(2.5)	1.0(1.3)	<0.01	1.8(1.7)	0.7	1.0
Improved a lot		2.8(3.3)	0.5(1.0)	<0.01	2.2(2.7)	0.7	0.8
p- value†					0.04		
**Parental version**							
Total score	0-24	4.0(5.9)	0.7(1.4)	<0,01	3.3(5.1)	0.6	0.7
No change		2.4(7.6)	0.5(1.6)	0.34	1.9(6.0)	0.3	0.3
Improved a little		2.7(5.3)	0.9(1.5)	0.02	1.8(4.1)	0.3	0.4
Improved a lot		4.6(5.9)	0.7(1.4)	<0.01	3.9(5.2)	0.7	0.7
p- value†					0.07		

ES = standardized effect sizes; SRM = standardized response mean.

*p-value derived from paired t-test.

†p-values derived from one-way analysis of variance.

For both versions of the SOHO-5, paired t-tests indicated that the mean scores in the pre- and post-treatment questionnaires of those who remained stable were not significantly different. Conversely, there was a significant difference in the pre- and post-treatment scores of those who reported improving a little (p < 0.05) and those who reported improving a lot (p < 0.001).

The MID is given by the mean change scores of those who reported improving a little. This study indicates that for the child-self report and parental report of the SOHO-5, this is equal to 1.8. As the standard deviation of change scores in stable children were 0.8 and 6.0 for child-self reports and parental reports, respectively, Guyatt’s responsiveness statistic equals 2.25 and 0.3.

### Longitudinal construct validity

Mean change scores in both versions showed a gradient in the expected direction across categories of the global transition judgment, however, the magnitude of change were tenuous (Table 2). The child-self report version showed good longitudinal construct validity due to the fact that those reporting improvement have positive change scores of increasing magnitude and those reporting stability have a change score close to zero. The negative mean change score in this latter category is due to one child with a score of −2 (indicating deterioration), while the remaining five children of the stable category had a change score of 0 (Table 3). Moreover, there were significant differences of mean change scores within categories of global transition judgments in the child self-report version (p = 0.04) (Table 2).

**Table 3:** Distribution of change scores for those who remained stable, improved a little and improved a lot (n = 154)

Change scores*	**‘Stayed the same’ (n = 6)**	**‘Improved a little’ (n = 24)**	**‘Improved a lot’ (n = 124)**
**Child-self report version**			
−2	1	0	0
0	5	7	50
+1 to +3	0	13	44
> + 3	0	4	30
**Parental report version**	**‘Stayed the same’ (n = 10)**	**‘Improved a little’ (n = 33)**	**‘Improved a lot’ (n = 111)**
0	9	19	29
+1 to +5	0	12	59
+6 to +12	0	0	14
+13 to +18	0	1	3
≥ +19	1	1	6

*Change scores are calculated by subtracting post-treatments scores from pretreatment scores of the SOHO-5.

The parental version showed moderate longitudinal construct validity taking into consideration that those reporting improvement had positive change scores among them; there were higher mean change scores for parents reporting that their children improved a lot than for those reporting that their children improved a little (Table 2). However, the mean change scores did not show an increasing magnitude across the ‘no change’ and ‘improved a little’ categories. There were no significant differences of mean change scores within categories of global transition judgments in the parental version (p = 0.07) (Table 2). Table 3 showed the distribution of change scores by global transition judgment categories. It can be observed that there was an outlier with a change score of 19 in the ‘no change’ category of the parental version of the SOHO-5; which results in the change score increasing of this category. If this parent was excluded from the analysis, the difference of mean change scores within categories of global transition judgments was significant (p = 0.012).

## Discussion

OHRQoL measures can potentially be used in oral health surveys, clinical trials, studies evaluating the outcomes of dental care programs, and in clinical practice in terms of identifying need, selecting therapies and monitoring patient progress [29]. Depending on the use, different psychometric properties may be essential for an OHRQoL measure. In that respect, the assessment of responsiveness and longitudinal construct validity, in addition to the standard properties of validity and reliability, are essential for using a measure in evaluating interventions. The Brazilian SOHO-5 has been proved valid, reliable, reproducible [9] and responsive to change.

When considering the results of this study it is important to discuss the nature of the sample in which we tested the Brazilian SOHO-5 responsiveness. The sample consisted of children of 5 to 6 years old and their respective parents who sought the dental screening at USP. Despite being a prospective patient sample, with expected volume of impacts, the large majority of children and parents reported low levels of impacts at baseline, and even though the mean change scores significantly declined after treatment, for total scores and within-categories of improvement.

Furthermore, there are two major approaches to assess responsiveness and they can be categorized under two broader groups: distribution-based and anchor-based, also known as internal “internal responsiveness” and “external responsiveness”, respectively [21,30,31]. The former compare the scores on a measure prior to and following an intervention and it uses the ES and SRM. In the anchor-based method, subjective global transition judgments can act as the anchor (reference) point. The present study encompassed both major approaches considering that there does not appear to be a consensus in the literature on what constitutes a responsive measure nor, correspondently, how responsiveness should be quantified. However, the anchor-based method (external responsiveness) has meaning in a wider range of settings than the more context specific concept of internal responsiveness and, for that reason, is more accepted and represents the best option for assessing responsiveness and for selecting outcomes measures for clinical studies [31].

In this study, both versions of the SOHO-5 showed good internal and external responsiveness. First, the total SOHO-5 scores showed a large significant decrease following treatment. ES and SRM were relatively similar in terms of magnitude of change observed in both versions of the SOHO-5. In addition, both versions showed longitudinal construct validity expressed by the mean SOHO-5 change scores showing a gradient in the expected direction across the categories of the global transition judgment and significant differences between the change groups, though; in the parental version this was the case after excluding one outlier. However, the small number of subjects in the stable category for both versions may have influenced the longitudinal construct validity.

In this study we did not have children and parents reporting deterioration in children’s OHRQoL after treatment, precluding this analysis for that category. Nevertheless, we obtained children who improved a little and a lot and children who remained stable, and there are sufficient to facilitate assessment of changes. On the other hand, our study would have more benefited having children and parents reporting deterioration in children’s OHRQoL after treatment in order to better assess the longitudinal construct validity characteristics of this global transition judgment category.

Comparing our results with other studies assessing the responsiveness of young children’s OHRQoL instruments, we can observe that the English version of the Early Childhood Oral Health Impact Scale (ECOHIS) has demonstrated some limited ability to be very responsive to change due to low levels of impacts [17], whereas the Chinese version of the same instrument was responsive to dental treatment [18]. Both studies also followed internal and external responsiveness’s criteria in their methods indicating through their results that the same measure cannot necessarily have the same technical properties in different samples. Therefore, it is important to mention that future versions of the original SOHO-5 must been tested in terms of responsiveness before it use in clinical trials. Based on the results, the Brazilian SOHO-5 is an instrument available in Brazil for measuring the effectiveness of clinical interventions in order to select the one that provides a better quality of life for patients.

## Conclusion

Taken together, the results of this study suggest that the Brazilian SOHO-5 is responsive to change and can be used in clinical trials. Both the parental and the child versions presented satisfactory results, however the child-self report version performed better.

## Competing interests

The authors declare that they have no competing interests.

## Authors’ contributions

JA was responsible for the acquisition of data, assisted in the analysis and interpretation of data and drafted the manuscript; GT and AS were responsible for the conception and design of the study and critical manuscript review; TMA and SMP performed interpretation of data and statistical analysis and critical manuscript review; DPR performed the acquisition of data and was responsible for critical manuscript review; MB was responsible for the conception and design of the study and critical manuscript review. All the authors read and approved the final manuscript. The authors declare that they have no competing interests. All authors read and approved the final manuscript.

## References

[B1] Abanto J Carvalho TS Mendes FM Wanderley MT Bönecker M Raggio DP Impact of oral diseases and disorders on oral health-related quality of life of preschool children Commun Dent Oral Epidemiol 2011 39 105 114 10.1111/j.1600-0528.2010.00580.x 21029148

[B2] Aldrigui JM Abanto J Carvalho TS Mendes FM Wanderley MT Bönecker M Raggio DP Impact of traumatic dental injuries and malocclusions on quality of life of young children Health Qual Life Outcomes 2011 9 78 10.1186/1477-7525-9-78 21943368 PMC3186738

[B3] Wong HM McGrath CP King NM Lo EC Oral health-related quality of life in Hong Kong preschool children Caries Res 2011 45 370 376 10.1159/000330231 21822015

[B4] Gradella CM Bernabé E Bönecker M Oliveira LB Caries prevalence and severity, and quality of life in Brazilian 2- to 4-year-old children Community Dent Oral Epidemiol 2011 39 498 504 10.1111/j.1600-0528.2011.00625.x 21692751

[B5] Goettems ML Ardenghi TM Romano AR Demarco FF Torriani DD Influence of maternal dental anxiety on oral health-related quality of life of preschool children Qual Life Res 2011 20 951 959 10.1007/s11136-010-9816-0 21181500

[B6] Brasil. Ministério da Saúde Projeto SB Brasil 2010: Pesquisa Nacional de Saúde Bucal. Brasília: Ministério de Saúde 2010 http://189.28.128.100/dab/docs/geral/projeto_sb2010_relatorio_final.pdf

[B7] De Vasconcelos Cunha Bonini GA Marcenes W Oliveira LB Sheiham A Bönecker M Trends in the prevalence of traumatic dental injuries in Brazilian preschool children Dent Traumatol 2009 25 594 598 10.1111/j.1600-9657.2009.00826.x 19788423

[B8] Tsakos G Blair YI Yusuf H Wright W Watt RG Macpherson LMD Developing a new self-reported scale of oral health outcomes for 5-year-old children (SOHO-5) Health Qual Life Outcomes 2012 10 62 10.1186/1477-7525-10-62 22676710 PMC3413607

[B9] Abanto J Tsakos G Paiva SM Raggio DP Goursand D Bönecker M Cross-cultural adaptation and psychometric properties of the Brazilian version of the Scale of Oral Health Outcomes for-5-year-old children (SOHO-5) Health Qual Life Outcomes 2013 11 16 10.1186/1477-7525-11-16 23394292 PMC3600026

[B10] Guyatt G Walter S Norman G Measuring change over time: assessing the usefulness of evaluative instruments J Chron Dis 1987 2 171 178 3818871 10.1016/0021-9681(87)90069-5

[B11] Deyo R Patrick D Reproducibility and responsiveness of health status measures: statistics and strategies for evaluation Control Clin Trials 1991 12 142S 158S 10.1016/S0197-2456(05)80019-4 1663851

[B12] Guyatt G Osoba D Wu A Wyrwich K Norman G Methods to explain the significance of health status measures 2002 Hamilton, Ontario: Clinical Significance Consensus Meeting Group, Unpublished paper 10.4065/77.4.371 11936935

[B13] Malden PE Thomson WM Jokovic A Locker D Changes in parent-assessed oral health-related quality of life among young children following dental treatment under general anaesthetic Community Dent Oral Epidemiol 2008 36 108 117 10.1111/j.1600-0528.2007.00374.x 18333874

[B14] Mashoto KO Astrom AN Skeie MS Masalu JR Changes in the quality of life of Tanzanian school children after treatment interventions using the Child-OIDP Eur J Oral Sci 2010 118 626 634 10.1111/j.1600-0722.2010.00776.x 21083625

[B15] Antunes LA Luiz RR Leão AT Maia LC Initial assessment of responsiveness of the P-CPQ (Brazilian Version) to describe the changes in quality of life after treatment for traumatic dental injury Dent Traumatol 2012 28 256 262 10.1111/j.1600-9657.2011.01094.x 22111923

[B16] Gaynor WN Thomson WM Changes in young children’s OHRQoL after dental treatment under general anaesthesia Int J Paediatr Dent 2012 22 258 264 10.1111/j.1365-263X.2011.01190.x 21999137

[B17] Li S Malkinson S Veronneau J Allison PJ Testing responsiveness to change for the early childhood oral health impact scale (ECOHIS) Community Dent Oral Epidemiol 2008 36 542 548 10.1111/j.1600-0528.2008.00434.x 18422706

[B18] Lee GHM McGrath C Yiu CKY King NM Sensitivity and responsiveness of the Chinese ECOHIS to dental treatment under general anaesthesia Community Dent Oral Epidemiol 2011 39 372 377 10.1111/j.1600-0528.2010.00604.x 21219374

[B19] Juniper G Guyatt G Willan A Griffith L Determining a minimal important change in a disease-specific quality of life questionnaire J Clin Epidemiol 1994 47 81 87 10.1016/0895-4356(94)90036-1 8283197

[B20] Jaeschke R Singer J Guyatt G Measurement of health status: ascertaining the minimal clinically important difference Control Clin Trials 1989 10 407 415 10.1016/0197-2456(89)90005-6 2691207

[B21] Tsakos G Allen PF Steele JG Locker D Interpreting oral health-related quality of life data Community Dent Oral Epidemiol 2001 40 193 200 22074311 10.1111/j.1600-0528.2011.00651.x

[B22] Beaton D Hogg-Johnson S Bombardier C Evaluating changes in health status. Reliability and responsiveness of five generic health status measures in workers with soft tissue injuries J Clin Epidemiol 1997 50 79 93 10.1016/S0895-4356(96)00296-X 9048693

[B23] Mackenzie C Charlson M DiGioia D Kelley K Can the sickness impact profile measure change? An example of scale assessment J Chronic Dis 1986 39 429 436 10.1016/0021-9681(86)90110-4 3711251

[B24] Locker D Jokovic A Clarke M Assessing the responsiveness of measures of oral health-related quality of life Community Dent Oral Epidemiol 2004 32 10 18 10.1111/j.1600-0528.2004.00114.x 14961835

[B25] Fitzpatrick R Ziebland S Jenkinson C Mowat A A comparison of the sensitivity to change of several health status measurements in rheumatoid arthritis J Rheumatol 1993 20 429 436 8478847

[B26] Cohen J Statistical power analysis for the behavioral sciences 1988 2 Hillsdale, NJ: Lawrence Erlbaum Associates

[B27] Liang MH Fossel AH Larson MG Comparisons of five health status instruments for orthopedic evaluation Med Care 1990 28 632 642 10.1097/00005650-199007000-00008 2366602

[B28] Juniper G Guyatt G Feeny D Ferrie P Griffith L Townsend M Measuring quality of life in asthma Qual Life Res 1996 5 35 46 10.1007/BF00435967 8901365

[B29] Locker D Applications of self-reported assessments of oral health outcomes J Dent Educ 1996 60 494 500 8675810

[B30] Revicki DA Hays RD Cella D Sloan J Recommended methods for determining responsiveness and minimally important differences for patient-reported outcomes J Clin Epidemiol 2008 61 102 109 10.1016/j.jclinepi.2007.03.012 18177782

[B31] Husted JA Cook RJ Farewell VT Gladman DD Methods for assessing responsiveness: a critical review and recommendations J Clin Epidemiol 2000 53 459 468 10.1016/S0895-4356(99)00206-1 10812317

